# PepString Server As a Tool to Search for Short Amino Acid Subsequences: Identification of Potential Amyloid-Beta Targets

**DOI:** 10.32607/actanaturae.27630

**Published:** 2025

**Authors:** S. A. Kozin, A. A. Anashkina, D. G. Matsuga, B. S. Suvaan, V. G. Tumanyan, V. A. Mitkevich, A. A. Makarov

**Affiliations:** Engelhardt Institute of Molecular Biology, Moscow, 119991 Russia; Biological Chemistry and Clinical laboratory diagnostics department, Astrakhan State Medical University, Astrakhan, 414000 Russia; Sechenov First Moscow State Medical University (Sechenov University), Moscow, 119991 Russia

**Keywords:** Alzheimer’s disease, amyloid-beta, short amino acid sequences, PepString, drug target, peptide, EVHH, HAEE

## Abstract

This paper presents a new bioinformatics tool to meet the needs of researchers in the search for short (≥ 3) amino acid subsequences in protein sequences annotated in public databases (UniprotKB, SwissProt) and illustrates its efficacy with the example of a search for the EVHH tetrapeptide in the human proteome, which is a molecular determinant of amyloid beta and is involved in interactions that are crucial in Alzheimer’s disease pathogenesis. The topicality of developing such a tool is, on the one hand, supported by experimental data on the role of short tetrapeptide motifs in the architecture of intermolecular interfaces. On the other hand, there are currently no software products for efficient search for short (≥3) amino acid sequences in public databases, which drastically limits researchers’ ability to identify proteins with exact matches of short subsequences. This tool (PepString server, http://pepstring.eimb.ru/) allows one to use intuitive queries to retrieve information about all the proteins that contain sequences of interest, as well as their combinations.

## INTRODUCTION

Protein-protein interactions play a fundamental role in virtually all cellular processes. Of particular interest to biomedicine and pharmaceuticals are protein- protein interfaces involving molecules associated with the development of pathological conditions. Neurodegenerative diseases are associated with aggregation of certain proteins into ordered supramolecular structures. In this case, the initiation of pathological aggregation occurs via a seeding mechanism. The key role in this mechanism is played by repetitive protein-protein interactions with identical intermolecular interfaces. One of the leading strategies for the development of disease-modifying drugs for the treatment of neurodegenerative diseases is the use of agents of various natures (e.g., antibodies, peptides, peptidomimetics) capable of specifically disrupting the formation of disease-associated intermolecular interfaces and thereby preventing unwanted aggregation [1]. Therefore, the identification of the amino acid residues that form these interfaces is crucially important.

Alzheimer’s disease (AD) is the most common neurodegenerative disease and the leading cause of dementia in the world [2]. AD is characterized by the conformational transformation of endogenous amyloid-β(Aβ) molecules from the monomeric state to soluble oligomers and insoluble aggregates [3] that initiate neuroinflammation and other pathological processes associated with AD development [4]. Insoluble Aβaggregates are present in the brain both as diffuse aggregates on the walls of blood vessels and as fibrillar aggregates (amyloid plaques) on the surface of neurons [5]. Aβaggregates and soluble oligomers are in dynamic equilibrium [6].

Aβ is a small polypeptide molecule consisting of 38–43 amino acid residues (aa) [7]. Aβ is produced by proteolysis of the amyloid precursor protein (APP) [8]. The amino acid sequence of the most abundant Aβ isoform in amyloid plaques, Aβ42, contains 42 aa [9, 10]. The Aβ peptide is present in both brain tissue and peripheral organs [11]. In the blood, Aβ exists mainly in platelets [12] and crosses the blood-brain barrier [11]. Aβ is found in the picomolar concentration range in the blood of both healthy individuals and sporadic AD patients [13]. The physiological functions of Aβ include suppression of microbial infections, regulation of synaptic plasticity, promotion of recovery after brain injury, sealing of the blood-brain barrier, and presumably suppression of tumor cell proliferation [14, 15].

Aggregation of Aβ molecules *in vivo *is initiated by intermolecular interactions. Zinc ions and the metalbinding domain (Aβ16) located in the 1–16 region of Aβ play a crucial role in these interactions. Therefore, data on the three-dimensional structure of Aβ16 and the molecular mechanism of zinc-dependent Aβ oligomerization are used for the rational search and design of candidate molecules on the basis of the anti-amyloid strategy [16]. The spatial structure of Aβ16 from several natural Aβ variants, in free and zinc-bound states, has been determined [17, 18, 19, 20, 21]. There are also experimental data on the structure of Aβ16 in amyloid fibrils isolated from the brain of AD patients [22]. According to these data, the 11-EVHH-14 region of human Aβ has a polypeptide backbone structure that remains almost unchanged both in the free Aβ16 molecule and in the complex of Aβ16 with the zinc ion, as well as in the N-terminal fragment of Aβ fibrils isolated from the brain tissue of AD patients.

Taken together, these properties characterize the 11-EVHH-14 site of Aβ as a structural invariant and suggest that this site plays an important role in the interaction of Aβ with other biological molecules. Indeed, the 11-EVHH-14 site of Aβ has been found (1) to be the main center for the recognition and binding of zinc ions, (2) to be located at the intermolecular interface in complexes between Aβ and the α4β2 subtype nicotinic acetylcholine receptor [23, 24], (3) to form a symmetric zinc-dependent interface in both Aβ dimers [25, 26] and Aβ oligomers [17], (4) to participate in zinc-dependent binding of nucleic acids [27]. The amino acid sequence of the Aβ metalbinding domain (Aβ16) is located in the extracellular membrane-spanning portion of the amyloid precursor protein (APP), it constitutes the C-terminal fragment of the soluble α-form of APP (sAPPα) [28], and both APP and sAPPα play vital physiological functions [29]. Furthermore, in both APP and sAPPα, the 11-EVHH-14 region of the Aβ metal-binding domain is sterically accessible for interactions with both zinc ions and other biomolecules, including Aβ. Thus, both of these proteins may act as potential binding partners for Aβ through zinc-dependent interactions via the symmetrical 11-EVHH-14 regions of the metalbinding domains in appropriate molecules.

In AD pathogenesis, the interaction of Aβ with zinc ions, mediated by the 11-EVHH-14 site of Aβ, is a key factor in the formation and spread of amyloid plaques. Therefore, this site is a promising drug target [16]. It is important to note that most of the monoclonal antibodies used to neutralize Aβ oligomers in AD therapy block the 11-EVHH-14 site [30]. However, monoclonal antibodies have many side effects [31]; so, the search for and development of low-molecular weight agents of various chemical classes [32], e.g., peptidomimetics and natural or artificial peptides [33], seems topical.

Recently, the use of a synthetic analogue of the 35-HAEE-38 site of the α4 subunit of the α4β2 subtype nicotinic acetylcholine receptor has been substantiated as an effective agent to inhibit the aggregation of endogenous Aβ molecules in AD pathogenesis [23]. This analogue (hereinafter HAEE) specifically binds to the 11-EVHH-14 site of Aβ both in the absence and in the presence of zinc ions, leading to the formation of stable complexes that, in turn, block the formation and propagation of Aβ aggregates [34]. However, it is unknown whether HAEE can bind to EVHH sites in other proteins, and how this may affect patients if HAEE is used as a drug. Given the key role of the EVHH tetrapeptide region in the formation of intermolecular interfaces involving Aβ, it is important to identify all proteins of the human proteome that contain this site because these proteins may be potential partners for Aβ. However, at the time of this study, there were no effective bioinformatics tools to search for short (≥ 3 aa) subsequences in known proteins. In this paper, we describe an original PepString server that could be used to search for exact matching of short protein sequence fragments. The use of the PepString server resources is illustrated with the example of the EVHH sequence of amyloid-beta, which is a promising target for the development of pathogenetic anti-amyloid drugs for AD treatment.

## EXPERIMENTAL PART

**PepString server**

**Fig. 1:**
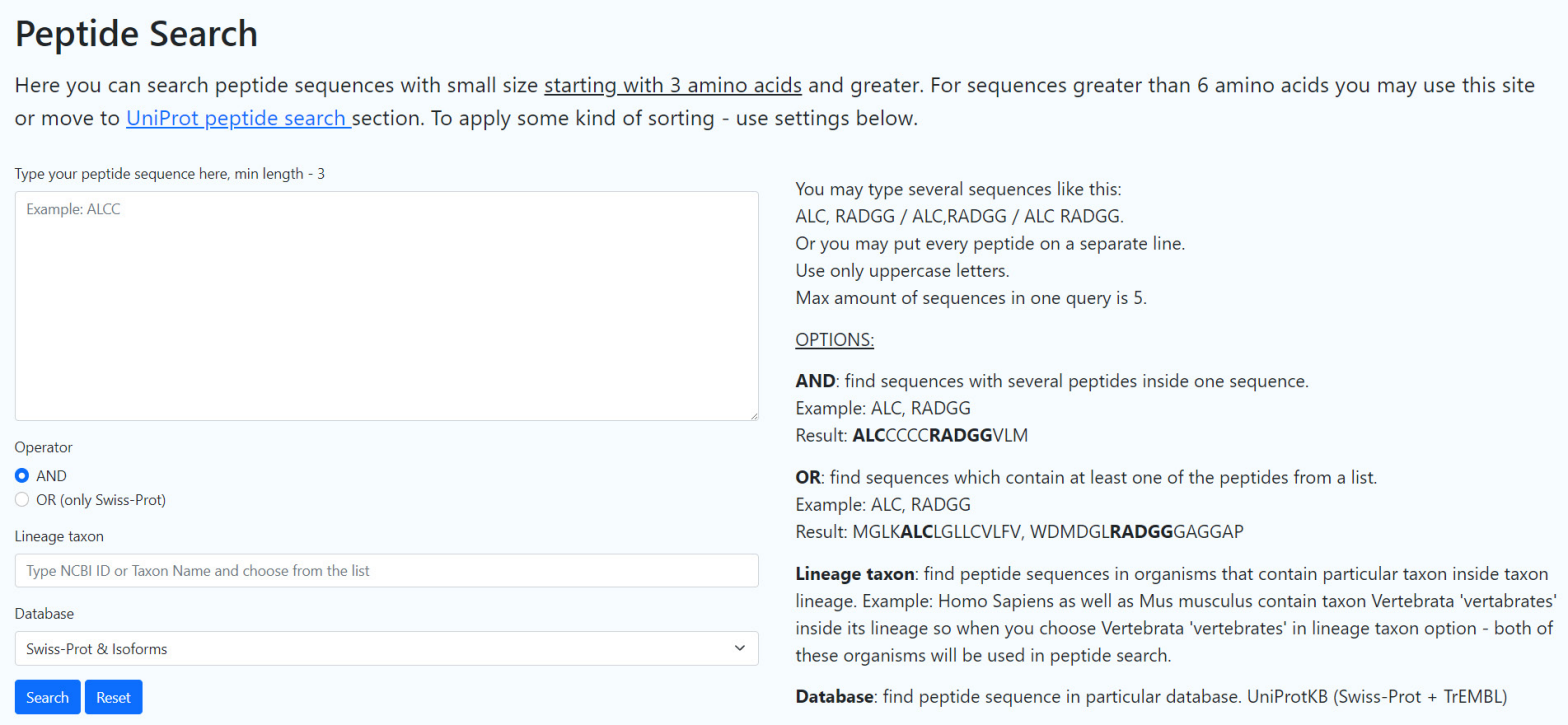
Query form to search for protein sequences containing exact matches of short peptides using the PepString server (http://pepstring.eimb.ru)

The PepString server (http://pepstring.eimb.ru) is developed based on the PostgreSQL version of the popular UniprotKB and SwissProt databases. This server allows the user to search for exact matches of short peptides in protein sequences. The query can be limited to a specific taxon, such as Mammalia, Bacteria, or Vertebrata, or to all species. The query can also be based on the presence of multiple fragments in a single sequence (≤ 5) or on the presence of at least one fragment from the list in a sequence. A screenshot of the home page is provided in *Fig. 1*. Multiple sequences can be entered using a comma and/or space as a separator, e.g., “ALC,RADGG”, “ALC, RADGG”, or “ALC RADGG”. Each of the multiple sequences can be placed on a separate line. One query can contain up to five sequences. Two operators, AND and OR, can be used for a search. The AND operator finds protein sequences that include all peptides from the query. The OR operator finds protein sequences that include at least one peptide from the query list. The search can be limited by selecting a taxon of any level, e.g., either Vertebrata, or Archaea, or Mammalia, or* Homo sapiens*.

**Fig. 2:**
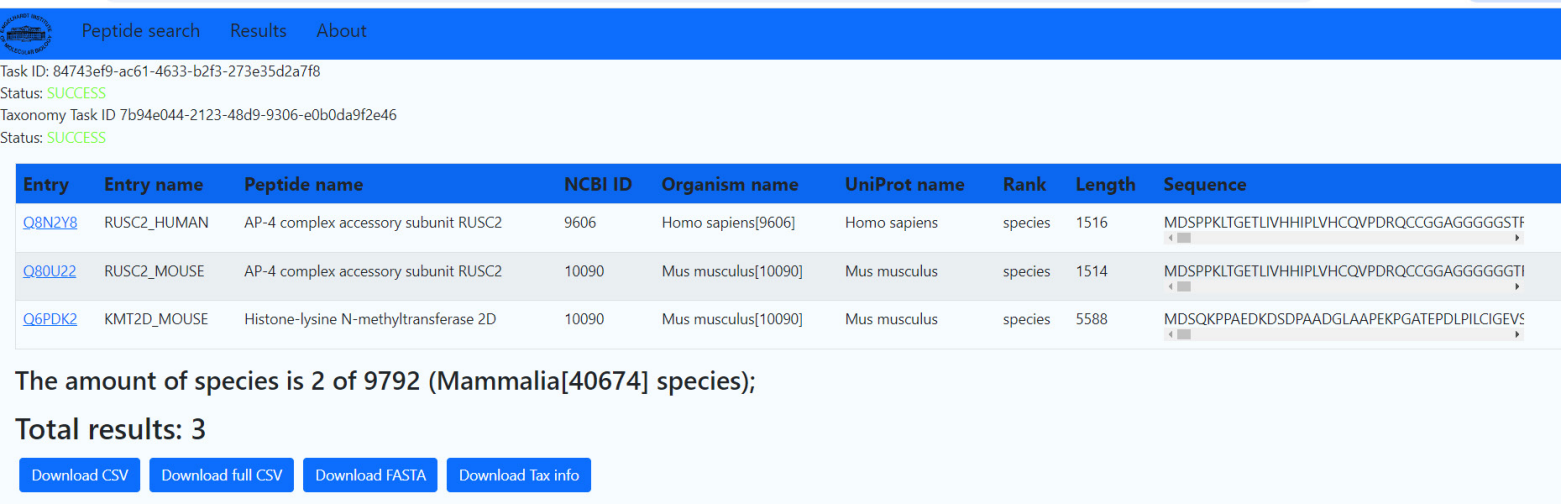
Search result for two ALC RADGG peptides among proteins from the Mammalia species using the PepString server

*Figure 2* shows an example of the results. The results are sorted by organism name. The user can save the query result in the FASTA or CSV format. Searching for a 3-amino acid fragment in the SwissProt database takes a few seconds, whereas in the UniprotKB database it takes from 10 minutes to several hours.

Python and PostgreSQL were used to create a protein sequence database based on UniprotKB (SwissProt+TrEMBL). The database structure diagram is shown in *Supplementary Fig. S1*. The database is updated automatically almost at the same time as the official UniProtKB database; i.e., approximately once every 8 weeks. The web interface is written using the Django framework.

The taxonomy in our database is identical to the NCBI taxonomy. Please note that NCBI identifiers and organism names may not match the data in the UniProt database. The reason is that UniProt also updates its taxonomy once every 8 weeks. However, our database uses the NCBI Taxonomy database that receives new updates daily, so the version we have used for the update may be different from the UniProt version.

The user’s query result is stored in the django_celery_ results_taskresult table for 24 h and then deleted.

**BLAST-based conservation calculations**

**Table 1:** Conservation of the EVHH fragment in human proteins determined by BLAST^*^

Conservation	Uniprot ID	Protein name	EVHH location
885/985 (89.8%)	P05067	Amyloid-beta precursor protein	682-685, YEVHHQ
683/800 (85.4%)	Q13634	Cadherin-18	46-49, TEVHHR
667/930 (71.7%)	P78310	Coxsackievirus and adenovirus receptor	272-275, KEVHHD
437/995 (43.9%)	Q06124	Tyrosine-protein phosphatase non-receptor type 11	441-444, EEVHHK
405/950 (42.6%)	Q9H8M1	Coenzyme Q-binding protein COQ10 homolog B	234-237, HEVHHT
276/965 (28.6%)	Q9Y2E6	E3 ubiquitin-protein ligase DTX4	575-578, NEVHHK
168/779 (21.6%)	Q03001-11	Neural isoform of dystonin	101-104, VEVHHQ
154/844 (18.2%)	Q58FF7	Putative heat shock protein HSP 90-beta-3	4-7, EEVHHG
133/859 (15.5%)	P08238	Heat shock protein HSP 90-beta	4-7, EEVHHG
134/990 (13.5%)	Q6ZSZ5	Rho guanine nucleotide exchange factor 18	459-462, TEVHHV
105/1000 (10.5%)	O75676	Ribosomal protein S6 kinase alpha-4	471-474, HEVHHD
100/991 (10.1%)	Q86SQ4	Adhesion G-protein coupled receptor G6	797-800, QEVHHP
81/872 (9.6%)	Q7Z3D6	D-glutamate cyclase, mitochondrial	273-276, PEVHHI
47/522 (9.0%)	Q9Y4G2	Pleckstrin homology domain-containing family M member 1	233-236, IEVHHS
87/974 (8.9%)	Q53F39	Metallophosphoesterase 1	309-312, CEVHHG
60/1000 (6.0%)	P54296	Myomesin-2	751-754, REVHHK
48/959 (5.0%)	O76064	E3 ubiquitin-protein ligase RNF8	229-232, TEVHHE
48/1000 (4.8%)	Q5TG30	Rho GTPase-activating protein 40	392-395, DEVHHN
43/998 (4.3%)	Q2M3C7	A-kinase anchor protein SPHKAP	682-685, DEVHHK
25/960 (2.7%)	P10912	Growth hormone receptor	71-74, DEVHHG
26/999 (2.6%)	Q8NH48	Olfactory receptor 5B3	171-174, NEVHHF
4/262 (1.5%)	Q9H0D2	Zinc finger protein 541	218-221, YEVHHG
9/949 (0.9%)	Q5VT97	Rho GTPase-activating protein SYDE2	567-570, REVHHT
4/998 (0.4%)	P41226	Ubiquitin-like modifier-activating enzyme 7	283-286, QEVHHA

^*^The entries are listed in descending order of conservation.

EVHH site conservation was calculated using the homologous protein sequences of other species from gnathostome vertebrates via the BLAST program [35]. Thousands of homologous sequences were found for each Uniprot protein identifier from *Table 1*. The number of sequences containing the EVHH site (the first number in the Conservation column in *Table 1*) was divided by the number of sequences with all variants of this site (the second number in the Conservation column in *Table 1*) and multiplied by 100 to obtain the Conservation value in %.

**EVHH site variants**

A short C++ program was written to parse the fasta sequence files and count all EVHH site variants. The program text is available upon request.

## RESULTS AND DISCUSSION

The PepString server found 63 sequences of 24 protein isoforms containing the EVHH fragment in the human proteome. If we take 1,000 homologous proteins from the Uniprot database and count how many of them contain a fragment homologous to EVHH, and in how many cases this fragment is exactly EVHH, we can form some idea of the conservation of this fragment in proteins in different species. In *Table 1*, we collected information on the EVHH fragment conservation in protein sequences and the position of this fragment in the sequence. A longer version of the table which lists all isoforms containing the EVHH fragment and structural information is presented in *Supplementary Table S1*. The EVHH fragment in the APP protein is the most conserved (89.8%) (*Table 1*). EVHH sites in the cadherin-18 and coxsackie virus and adenovirus receptor sequences are somewhat less conserved, 85.4 and 71.7%, respectively. The neuronal isoform of dystonin also deserves attention, because, in addition to the conserved form of EVHH found in 168 out of 779 sequences (21.6%), 315 out of 779 sequences (40.4%) of homologous proteins from different species contain a sequence of this EAHH site that is very similar in physicochemical properties.

**Table 2:** EVHH site variants found in homologues of human APP (P05067) from different species

Motif	Number of sequences	Species
EVHH	885	Balaenoptera acutorostrata scammoni (North Pacific minke whale), Balaenoptera musculus (Blue whale), Castor canadensis (American beaver), Chinchilla lanigera (Long-tailed chinchilla), Dipodomys ordii (Ord’s kangaroo rat), Fukomys damarensis (Damaraland mole rat), Haplochromis burtoni (Burton’s mouthbrooder), Heterocephalus glaber (Naked mole rat), Ictidomys tridecemlineatus (Thirteen-lined ground squirrel), Jaculus jaculus (Lesser Egyptian jerboa), Marmota marmota marmota (Alpine marmot), Mesocricetus auratus (Golden hamster), Microtus ochrogaster (Prairie vole), Mus musculus (Mouse), Mus spicilegus (Steppe mouse), Nannospalax galili (Northern Israeli blind subterranean mole rat), Octodon degus (Degu), Peromyscus maniculatus bairdii (Prairie deer mouse), Rattus norvegicus (Rat), Sciurus vulgaris (Eurasian red squirrel), Urocitellus parryii (Arctic ground squirrel), Pundamilia nyererei, Maylandia zebra (Zebra mbuna), Atractosteus spatula (Alligator gar), Lepisosteus oculatus (Spotted gar), Oreochromis aureus (Israeli tilapia), Oreochromis niloticus (Nile tilapia)
EVYH	24	Cyprinus carpio carpio, Cirrhinus molitorella (Mud carp), Onychostoma macrolepis, Sinocyclocheilus rhinocerous, Sinocyclocheilus anshuiensis, Danio rerio (Zebrafish), Sinocyclocheilus grahami (Dianchi golden-line fish), Triplophysa rosa (Cave loach)
AVHH	7	Oryzias javanicus (Javanese ricefish), Oryzias latipes (Japanese rice fish), Oryzias melastigma (Marine medaka), Oryzias sinensis (Chinese medaka)
-VHH	1	Clupea harengus (Atlantic herring)
EVHP	1	Denticeps clupeoides (Denticle herring)
EV-H	1	Astyanax mexicanus (Blind cave fish)
EVYP	1	Triplophysa tibetana
RGGW	1	Puma concolor (Mountain lion)

In the human amyloid precursor protein (APP) sequence, EVHH is located in the Aβ peptide and is able to bind with the Zn^2+^ ion and form dimers and oligomers [17, 21, 25]. Analysis of APP protein sequences from other gnathostome vertebrate species revealed that APP sequences in all birds, reptiles, amphibians, fishes, and almost all mammals contain a highly conserved variant of EVHH, with a few exceptions (*Table 2*). For example, both APP isoforms from blue and fin whales contain the EVRH sequence, although the conserved EVHH variant is present in other marine mammals. The same EVRH variant is found in some rodents, including mice, rats, moles, ground squirrels, degus, and naked mole rats (see *Table 2* for a complete list). The substitution is not found in all rodents. For example, we found the conserved EVHH variant in APP sequences from *Oryctolagus cuniculus* (rabbit) and *Chrysochloris asiatica *(Cape golden mole). Another exception is *Puma concolor *(mountain lion) that has a single APP isoform comprising a completely different RGGW site at this location.

Further, we will consider the functions of each identified protein. Cadherin-18 is annotated in Uniprot as a protein involved in calcium-dependent cell-cell adhesion, cell migration, and morphogenesis. In the cadherin-18 sequence, the EVHH fragment is located in the 25–53 propeptide that is cleaved from the protein during maturation. In the AlphaFold model of the structure of this protein, the EVHH site is located in a disordered loop, on the protein surface (*Supplementary, Table S1*). The function of this propeptide is not yet known. According to Uniprot, asparagine residue 36 can be glycosylated, which makes the propeptide sensitive to blood glucose levels. Cadherin-18 is known to be associated with AD. Cadherin was experimentally shown to interact with presenilin-1 that is involved in AD [36].

The coxsackievirus and adenovirus receptor is a component of the epithelial apical junction complex, which can function as a homophilic cell adhesion molecule and is required for maintaining tight junctions [37]. It is also involved in transepithelial leukocyte migration through adhesive interactions with JAML, a transmembrane protein of the plasma membrane of leukocytes. After binding to the epithelial coxsackievirus adenovirus receptor (CXADR), JAML induces subsequent signaling events in gamma-delta T cells through PI3-kinase and MAP-kinase. This leads to T cell proliferation and production of cytokines and growth factors that, in turn, stimulate epithelial tissue repair [38]. The EVHH fragment is located in the 269–285 domain of this receptor that is annotated as a domain rich in charged amino acids. This means that this fragment is very flexible and can change its conformation depending on the structure of the interaction partner. The coxsackievirus and adenovirus receptor is associated with AD. Coxsackievirus [39] or adenovirus [40] infection has been shown to be capable of triggering the onset of AD in the elderly, because it provokes prion protein expression.

The next protein with high conservation of the EVHH fragment is tyrosine phosphatase non-receptor type 11 that is involved in cascades of various receptor and cytoplasmic tyrosine kinases and participates in signal transmission from the cell surface to the nucleus. Kinase activation suppresses the function of integrins and causes dephosphorylation of focal adhesion kinase [41]. It is one of the important negative regulators of the nuclear export of telomerase reverse transcriptase [42]. Mutations in this protein are associated with a number of diseases, e.g., LEOPARD syndrome [43] or Noonan syndrome [44], that develop due to downregulation of the intracellular RAS/MAPK signaling pathway. There are currently no data on any association with AD.

The mitochondrial coenzyme Q-binding protein COQ10 homologue B (Q9H8M1) is necessary for the function of coenzyme Q10 in the respiratory chain and may serve as a chaperone or may participate in the transport of Q10 from its site of synthesis to the catalytic sites of the respiratory complexes. According to the AlphaFold model, the EVHH site is a part of the β-strand on the protein surface (see* Supplementary*, *Table S1*). There is an opinion in the scientific community that the introduction of coenzyme Q10 increases the concentration of mitochondria in the brain and provides a neuroprotective capability [45, 46]. However, this was not convincingly shown in phase II clinical trials, and it was decided not to conduct phase III clinical trials [47].

E3 ubiquitin-protein ligase DTX4 (Q9Y2E6) is involved in the negative regulation of type I interferon signaling through NLRP4 by targeting the kinase TBK1 for degradation [48]. In addition to 276/965 (28.6%) identified conserved EVHH sequences, an EIHH fragment with very similar physicochemical properties was found in 687/965 (71.2%) sequences. A homologous ubiquitin ligase DTX2 is associated with small vessel damage in the early stages of AD [49].

A neuronal isoform of dystonin (Q03001-11), apart from an EVHH site variant found in 168 out of 779 sequences (21.6%), also occurs as a very physicochemically similar EAHH site variant in 315 out of 779 sequences (40.4%). Mutations in the gene for this protein lead to progressive degeneration of sensory neurons in mice. These mice suffer from sensory ataxia and die by weaning age [50]. They develop a severe movement disorder due to sensory neuron degeneration [51].

**Fig. 3:**
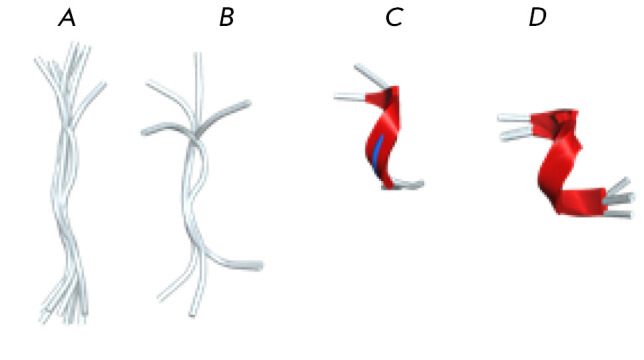
EVHH site conformations in human protein structures form four clusters. (A) Unfolded conformation (O76064, Q9H8M1, Q5VT97, Q13634, Q7Z3D6, Q9Y4G2, P08238, Q58FF7, P10912, O75676, Q53F39). (B) Unstructured conformation (Q9Y2E6, Q5TG30, Q8NH48, Q86SQ4). (C) Convoluted conformation (P54296, 1ze9). (D) α-Helix (Q2M3C7 (EVHH site within the globule), Q9H0D2, 3B7O, P78310, Q6ZSZ5 (EVHH site within the globule), P41226). Only EVHH sites in Q6ZSZ5 and Q2M3C7 are buried in the protein globule and are inaccessible

We analyzed EVHH site locations and conformations in protein structures. In human proteins, EVHH site conformations form four clusters (*Fig. 3*). The conformation of the EVHH site in myomesin is the closest to that of the zinc-binding domain in Aβ. An association of myomesin-2 with AD was also found. Investigation of cardiomyopathy in transgenic mice showed that small heat shock protein α-B-crystallin (CryAB) aggregates found in diseased hearts contained an amyloid oligomer that may be the main toxic species in AD and other amyloid-associated degenerative diseases [52]. α-B-crystallin is known to interact with myomesin-2 [53].

In 21 out of 24 structures, the EVHH site is located on the protein surface. There is no AlphaFold model for the dystonin sequence (Q03001-11). In the Rho guanine nucleotide exchange factor 18 (Q6ZSZ5) model, the EVHH site is located inside the protein globule. In the A-kinase anchor protein SPHKAP (Q2M3C7) model, the EVHH site is surrounded by unstructured loops.

In summary, the 11-EVHH-14 site in the Aβ sequence is highly conserved in all gnathostome vertebrates. Gnanthostomes amount to more than 99% of all living vertebrate species, including humans. Previously, the H13R substitution was shown to protect rats from AD [18]. In *Balaenoptera acutorostrata scammoni *(North Pacific minke whale) and* Balaenoptera musculus *(blue whale), as well as in some rodents, such as *Heterocephalus glaber *(naked mole rat) or *Nannospalax galili *(Northern Israeli blind subterranean mole rat) (*Table 2*), exactly this substitution that converts EVHH into EVRH exists, which apparently renders these species protected against pathological Aβ aggregation and thus not susceptible to AD. The explanation for this follows from the molecular mechanism of Zn-dependent oligomerization of Aβ [17], which indicates a key role for the EVHH site in the pathological process.

We found that the EVHH site was present in 63 isoforms of 24 proteins. Each of these proteins may be a potential molecular partner of zinc-dependent interaction with the amyloid-beta molecule. Some of them are known to be associated with AD pathogenesis, but there is no data on the mechanisms of their action. Six of the 24 identified proteins, namely APP, cadherin-18, coxsackievirus and adenovirus receptor, adhesion G protein-coupled receptor G6, growth hormone receptor, and olfactory receptor 5B3, reside in the cell membrane, are receptors, and probably transmit a signal into the cell.

The identified proteins are both potential targets for HAEE and possible partners of Aβ. As shown previously [17, 25], residues of site 11-14 of the Aβ peptide (EVHH) form a zinc-mediated interface with a similar region of another Aβ molecule. It is logical to assume that such interactions can occur not only between identical molecules of amyloid-beta, but also with other proteins that have a similar region available for interaction.

Let us make several suggestions. First, APP functions as a cell surface receptor and performs physiological functions on the surface of neurons, which are related to neurite outgrowth, neuronal adhesion, and axonogenesis [54]. Interactions between APP molecules on neighboring cells are known to promote synaptogenesis [54]. Since zinc ions are involved in synaptogenesis, we venture to suggest that the interaction between APP molecules occurs through the Zn-dependent interface of EVHH sites. This bold suggestion requires further experimental evidence.

EVHH sites have been found in mitochondrial proteins, D-glutamate cyclase, and coenzyme Q-binding protein Q9H8M1. Since Aβ peptide is known to induce the AGER-dependent pathway that involves activation of p38 MAPK, resulting in internalization of the Aβ peptide and mitochondrial dysfunction in cultured cortical neurons [55], the second suggestion is that the Aβ peptide is able to penetrate the mitochondrial membrane and form zinc-dependent complexes with one or both proteins.

Notably, another of the identified EVHH site-containing proteins, namely tyrosine-protein phosphatase non-receptor type 11, positively regulates the MAPK signaling pathway [44]. A third suggestion is that Aβ regulates the MAPK signal transduction pathway through the zinc-dependent interface with tyrosineprotein phosphatase non-receptor type 11. Another protein from this list, namely the coxsackievirus and adenovirus receptor, also triggers one of the MAPK activation pathways.

Another group of proteins, which we found to be involved in the regulation of neuronal activity, includes cadherin-18, coxsackievirus and adenovirus receptor, and adhesion G-protein-coupled receptor G6 that interacts with laminin-2, Rho guanine nucleotide exchange factor 18, and dystonin. Cadherins are calcium-dependent cell adhesion proteins. They preferentially interact amongst themselves in a homophilic manner in connecting cells; thus, cadherins may facilitate the sorting of heterogeneous cell types. The coxsackievirus and adenovirus receptor, in addition to its negative role in virus entry, is a component of the epithelial apical–junctional complex, which can function as a homophilic cell adhesion molecule and is essential to the integrity of tight junctions. The adhesion G-protein-coupled receptor G6 is a major component of the basal membrane. It couples with G(i) and G(s) proteins and is required for normal differentiation of promyelinating Schwann cells and for normal axonal myelination [56]. Rho factor 18 acts as a guanine nucleotide exchange factor (GEF) for the GTPase RhoA, inducing the formation of actin stress fibers and the production of reactive oxygen species (ROS). It can be activated by the beta-gamma subunits of G proteins [57]. The neuronal isoform of dystonin is poorly understood. Mutations in the gene for this protein in mice are known to result in progressive degeneration of sensory neurons. These mice suffer from sensory ataxia and die by weaning age [50]. The fourth suggestion is that these proteins are partners of the G protein, and the interaction with them through the zinc-dependent interface affects the function of the G protein and G protein-associated processes in the cell. However, the structural model of Rho guanine nucleotide exchange factor 18 suggests that the EVHH site is located inside the protein globule and is inaccessible to a solvent.

The fifth suggestion is that the Aβ peptide can form complexes with two heat shock proteins, HSP 90-beta (P08238) and 90-beta-3 (Q58FF7), through the zinc-dependent interface and affects maturation, maintenance of the structure, and proper regulation of specific target proteins. Apart from chaperone activity, it also plays a role in the regulation of the transcription mechanism. HSP90 and its co-chaperones modulate transcription at least on three different levels. First, they alter the steady-state levels of certain transcription factors in response to various physiological signals. Second, they modulate the activity of some epigenetic modifiers, such as histone deacetylases or DNA methyltransferases, and respond to changes in the environment. Third, they are involved in the migration of histones from the promoter region of certain genes and, thereby, switch on gene expression [58].

Gene expression can also be influenced by zinc finger protein 541 (Q9H0D2). This transcriptional regulator is essential for male fertility and meiotic prophase completion in spermatocytes. The aforementioned tyrosine-protein phosphatase non-receptor type 11 (Q06124) is also involved in a cascade of various receptor and cytoplasmic protein tyrosine kinases, participating in signal transmission from the cell surface to the nucleus. Zinc finger proteins [59] and tyrosine phosphatases [60] are known to be associated with AD, but the exact mechanism of the interaction remains unknown.

## CONCLUSION

This paper introduces an original PepString server for the search for short amino acid sequences in the UniprotKB and SwissProt databases. Using the PepString server, we demonstrated for the first time that the tetrapeptide EVHH site, which is a structural and functional determinant of human amyloid-beta both in health and in AD, is present in 63 isoforms of 24 proteins. On the basis of an analysis of data on the association between these proteins and AD, we proposed a potential role for cadherin-18, coxsackievirus and adenovirus receptor, E3-ubiquitin ligase DTX4, the neuronal isoform of dystonin, and myomesin-2 in AD pathogenesis.
